# Perspectives on Quality of Life in Glaucoma

**DOI:** 10.5005/jp-journals-10008-1101

**Published:** 2012-10-16

**Authors:** Divjyot Kaur, Anita Gupta, Gursatinder Singh

**Affiliations:** 1 Junior Resident, Department of Pharmacology, Government Medical College, Patiala, Punjab, India; 2 Professor and Head, Department of Pharmacology, Government Medical College, Patiala, Punjab, India; 3 Associate Professor, Department of Pharmacology, Government Medical College, Patiala, Punjab, India

**Keywords:** Glaucoma, Chronic disease, Asymptomatic, Quality of life, Patient reported outcome, Compliance.

## Abstract

Chronic diseases are invariably associated with decreased functioning ability of the individual in one form or the other depending upon the system/organ involved. Disability consequent to the disease is the major factor affecting the patient’s physical and psychosocial well-being; in other words, the ‘Quality of Life (QOL)’. Besides the disease itself, the treatment and its consequences are also major determinants of QOL of the patients. Globally, glaucoma, which is emerging as one of the leading causes of blindness, is one such chronic ophthalmic disease characterized by a progressive loss of visual function and a potential to cause irreversible blindness, if not treated at an early stage. Patients of glaucoma need to take lifelong medications in order to keep their intraocular pressure within limits. It’s impact on the daily life of patients cannot be overexpressed and compounded by the fact that it remains asymptomatic for a considerable time after the disease has set in; has led to new imperatives in diagnosis, treatment and epidemiological and outcome studies. Assessment of the debilitating effect of glaucoma and side effects of its treatment on the emotional and physical QOL of the patient is therefore an important criterion for arriving at the treatment regimen. An extensive literature search was done on Pubmed Central, Pubmed and Google Scholar using the keywords ‘glaucoma’, ‘quality of life in glaucoma’, ‘management in POAG’ and ‘QOL assessment tools’. Various tools available for the assessment of QOL, and their advantages and limitations have been reviewed in this article.

## INTRODUCTION

WHO defines quality of life (QOL) as ‘patients’ perspective of their position in life in the context of the culture and value systems in which they live and in relation to their goals, expectations, standards and concerns’.^1^ This definition focuses upon the respondents’ ‘perceived’ quality of life and, thus, measures the effects of both, the disease and health interventions, on the patients’ quality of life.

### Disease Magnitude

Glaucoma is the second leading cause of blindness worldwide, with an estimated 12 million patients in India,^2^ and over 60 million people affected by it worldwide.^3^ It is a chronic progressive optic neuropathy resulting from slow progressive degeneration of retinal ganglion cells and optic nerve axons.^4,5^ The diagnosis and treatment is often delayed because (i) glaucoma is painless and (ii) the patient remains asymptomatic for a long time during the disease progression in early stages. The late manifestations of visual symptoms are essentially due to the pattern of visual field involvement wherein the mid peripheral vision is affected first followed by central vision and then fixation. It is only when the visual field loss involves the central vision, does the patient become aware of a functional defect.^6^ Large proportion of patient population at risk is from the elderly age group and they generally ignore it as an age related inevitability.^3^ By the time, the patient experiences symptoms of visual impairment, the disease usually has reached an advanced stage wherein irreversible damage has already occurred. So, early diagnosis and treatment plays a very important role in the management of glaucoma.^3,6^

### Goal of Treatment

The efficacy of the treatment by convention is evaluated by clinical indicators, such as intraocular pressure levels, Snellen visual acuity, perimetric findings and side effects of treatment, which are essentially objective measures of assessment. However, the nature of this disability leads to physical, psychological and social dysfunction and its impact is individual specific. The goal of glaucoma therapy is not only to control or reduce the IOP to a target level but also to ensure that the patient has a good functional vision for the duration of his/her lifetime, while maintaining a good quality of life. Today, patient-reported outcomes (subjective measures) are becoming increasingly important criteria to evaluate treatment efficacy.^7-9^

Currently, the mainstay of treatment is reduction of intraocular pressure (IOP) as it appears to be the only modifiable risk factor for glaucoma.^10^ The IOP control helps in preventing damage to the optic nerve. This is achieved by laser therapy, surgery or medications. First line treatment of glaucoma involves the use of medications which may have to be continued for lifetime. This can be either a single drug or a combination of drugs to be instilled into the eyes. Usually, it is only when these medications fail to reduce or control the IOP, that a laser or surgical intervention is done.^4,11^

### Impact of Disease on QOL

The impact of glaucoma on QOL is primarily due to (i) psychological factor―the fact that one is suffering from a potentially blinding chronic disease causes anxiety and fear in patients and their families, (ii) functional disability due to the disease, (iii) medication side effects and inconvenience and (iv) cost of treatment (impact on livelihood).^12,13^

Functional disability due to this disease is primarily because of difficulties experienced by the patients in performing vision related activities of daily life. These include activities, like reading, driving, walking, climbing downstairs, various household chores, like sewing, cooking, fixing, etc. and limitations in social relations because of vision problems. In addition, an individual’s perception of his or her visual function may vary depending upon a number of factors ranging from perceptions about what is ‘normal’ for a certain age, issues related to ‘socially desirable’ response, to apparent failures to perform certain visual tasks successfully. This was seen clearly in the collaborative initial glaucoma treatment study (CIGTS), where age was found to be a significant factor in determining QOL, indicating that younger respondents reported more problems than the older age group. This was probably because older adults have lower expectations of their health status. Gender was also found to be an important factor with females reporting more problems than males. In addition to this, patients with lower income reported more problems.^14,15^

### Problems with Medications and Their Impact on QOL

There are certain limitations associated with the use of medications, like, inconvenience of use of multiple drugs and multiple doses in a day; and difficulty in self-administering eye drops by elderly patients. Additionally, the use of eye drops is associated with side effects, e.g. burning sensation, redness in the eye (carbonic anhydrase inhibitors), bronchospasm and bradycardia (beta blockers), iris pigmentation and increase in length of eyelashes (as with prostaglandin analogues), etc.^5,11^ Use of topical antiglaucoma drugs has been associated with a higher incidence of dry eye syndrome (an ocular surface disease). This has been attributed to the interaction of the active agent itself or the preservatives used with the ocular surface tissues. Benzalkonium chloride (BAK) is a detergent-like preservative common to most anti-glaucoma medications. It has been found to cause damage to the corneal and conjuctival epithelial cells, and decrease conjunctival goblet cell density, thus reducing tear film stability and promoting dry eye.^16,17^ Presence of dry eye syndrome (DES) has a negative impact on the patient’s QOL.^16,18^ Cost of treatment is another bothersome aspect that has to be taken into consideration from the patient’s perspective. All these factors add up to consequent poor patient compliance, which in turn lowers the effectiveness of treatment; ultimately increasing the cost burden on health care system.^5,11^

### Compliance and QOL

The risk of noncompliance in chronic disease is always high; and its impact on glaucoma can be more serious as it may lead to irreversible blindness and poor QOL.^19^ Robin AL et al conducted a study to examine the effect of adding complexity to a glaucoma medical treatment regimen―specifically, what would occur to the refill rate (and, by inference, to adherence), when a second medication was added to a currently used once-daily drug. The results showed that with the addition of a second medication, there was a statistically and clinically significant increase in the refill interval (i.e. decreased adherence) which in turn may affect intraocular pressure control due to the medication-free periods between refills.^20^

In another study conducted by Nordmann et al, the relationship between vision-related quality of life and local side effects with anti-glaucoma drugs was evaluated on a representative French sample of glaucoma patients. Based on the study results, it was concluded that vision-related QOL was poor in patients with topical drug-induced local side effects, which led to poor patient satisfaction and in turn, a poor compliance. Additionally, poor patient satisfaction was linked to increased number of visits to the ophthalmologist.^21^ Compliance and adherence to therapy being crucial for optimum long-term outcome, it is very important to keep the treatment regimen as simple as possible, comprising of the use of maximally effective medications with least ocular and systemic side effects and at affordable cost to the patient.^10^ A proper evaluation of QOL in glaucoma patients will, thus, help in arriving at the specifically tailored treatment regimen for a given patient.

### QOL Assessment Tools

Various tools (in the form of questionnaires) are available for measuring QOL and are classified in Table 1.^22,23^ These questionnaires are of two types; self-administered questionnaires and those administered by a trained technician either directly or by telephone.^1^

While there is no gold standard QOL assessment scale, glaucoma-specific and vision-specific instruments are better than generic tools to assess the impact of the disease *per se* on the patients’ overall well-being.^22-24^ This has been seen in various studies, e.g. a study was conducted by Lester M et al to evaluate the QOL in glaucomatous patients using two different questionnaires: the medical outcomes study 36-item short form health survey (MOS SF-36) (generic tool) and Viswanathan et al’s questionnaire (glaucoma-specific tool) and to compare these two questionnaires. Results of their study showed that Viswanathan et al’s questionnaire was more significantly correlated to visual field indices (p < 0.0001 as against p < 0.05 with SF-36 scale) and was a much more sensitive tool in assessment of QOL in glaucomatous patients.^13^

Similarly, Parrish RK, in his study to determine the relation between visual impairment, visual functioning and the global quality of life in patients with glaucoma, concluded that SF-36 (generic tool) is unlikely to be useful in determining visual impairment in patients with glaucoma as it had a weak correlation. On the other hand, there was a moderate correlation between visual field impairment and VF-14 score which showed that it may be generalizable to patients with glaucoma. Moreover, several of the NEI-VFQ scales correlated with visual field impairment scores in patients with a wide range of glaucomatous damage.^25^

There is also availability of tools like comparison of ophthalmic medications for tolerability (COMTOL) which can assess the treatment satisfaction of patients.^11,23,24^ COMTOL questionnaire was developed by Barber et al to compare the tolerability of topical ophthalmic medications used in treatment of glaucoma. It was designed to capture the frequency and bother of common side effects (ocular and other local effects and effects on visual function) of topical drugs used to control intraocular pressure. The questionnaire also measures the extent to which these side effects and any associated limitations in routine living activities interfere with health-related quality of life, medication compliance and patient satisfaction with the medication. Moreover, in their study, the COMTOL questionnaire showed acceptable measurement characteristics for inclusion as a tolerability measure to supplement spontaneous adverse event reporting in clinical trials of topical ophthalmic therapy.^24^ To date, COMTOL is the only valid measure of treatment satisfaction with medication (TS-M) for ocular hypotensive medications.^11^

**Table 1:** **Table 1:** Various instruments/tools/questionnaires available to measure QOL

*Tools for assessment of QOL*		*Examples*	
1. Generic/general health instruments		• Sickness impact profile (SIP) • Medical outcomes study short form-36 (SF-36) • MOS-20	
a. Utility measures		• Time trade-off • Thermometer • Choice-based conjoint analysis	
2. Specific instruments			
a. Vision-specific instruments		• The national eye institute visual function questionnaire (NEI-VFQ) 51 item and (NEI VFQ-25) 25 item • Activities of daily vision scale (ADVS) • VF-14 • VAQ	
b. Glaucoma-specific instruments		• Glaucoma symptom scale (GSS) • Glaucoma quality of life-15 (GQL-15) • Viswanathan et al • SIG (symptom impact glaucoma score) • GHPI (Glaucoma health perceptions index)	
c. Treatment-specific instruments		• Comparison of ophthalmic medication for tolerability (COMTOL)	

### Limitations of Existing Instruments

There are certain limitations associated with the presently available tools. Although, the specific instruments are far more sensitive and specific than generic instruments with respect to ophthalmic problems, all except the NEI-VFQ provide little information on the general status of the patient. These scales do not take the patient’s age into account. Moreover, no specific scale has been developed for assessment in children and adolescents, although the impact of vision on daily life is important in them as well. In addition, many vision-specific tools have the inadequacy to capture certain important issues like peripheral and color vision which are also affected by glaucoma.^26,27^ COMTOL questionnaire suffers from the inadequacy of not covering the side effects of prostaglandin analogs, as their use was not widespread back in 1996.^11^ Today, these are the preferred first line agents for glaucoma.^5,28^

Thus, there is a need to improve these tools from time to time to arrive at patient specific QOL measure; improving patient’s understanding of their own condition through dialogue and counseling by the clinician; formulating a proper treatment regimen for the individual patient; and to ensure compliance which is the key to preserving vision and independence.

Thus, with the assessment of patients’ QOL, the clinician can be better informed about the patients’ perspective of their disease condition, their convenience with the treatment (both side effects and cost); and their compliance and adherence to the medications. Not only will this help, the ophthalmologist make better decisions regarding further treatment regimen, but patients might also prove to be more active participants in the decision-making process related to the various therapeutic options available for treatment of their disease.^7^ These tools, therefore, need to be more user-friendly so that they can be more easily and routinely administered by the clinicians.^23^ However, in order to bring about an improvement in these instruments, it is essential that the practitioners continue to use and test the available tools, thus generating more specific instruments with regard to pathologies or particular population subgroups.^27^

## CONCLUSION

Glaucoma, a debilitating, chronic and a potentially blinding disease of the eye, brings about major limitations in day-to-day activities in the individual. Early diagnosis and treatment have a vital role in preventing functional damage of vision from this dreaded disease. Since maximization of patients’ QOL is the aim of any clinical intervention,^29^ assessment of QOL of patients is important in order to be able to provide them with the best suitable and convenient treatment possible. With the use of QOL assessment tools, treatment can be individualized in a better way as it helps the clinician in striking a balance between benefits and risks of the treatment concerned. It also helps in recognizing the possible obstacles to patient compliance early in the treatment plan, in turn resulting in a more effective and successful control of disease progression. Ophthalmologists should try and make a greater use of such tools in their practice in order to be able to provide their patients with the best possible treatment not only in terms of vision (controlling further damage to the optic nerve) but also by maintaining or improving their overall quality of life.
